# Making assembly line in supply chain robust and secure using UHF RFID

**DOI:** 10.1038/s41598-021-97598-5

**Published:** 2021-09-10

**Authors:** Abubakar Sharif, Rajesh Kumar, Jun Ouyang, Hasan T. Abbas, Akram Alomainy, Kamran Arshad, Khaled Assaleh, Ayman Althuwayb, Muhammad Ali Imran, Qammer H. Abbasi

**Affiliations:** 1 School of Electronic Science and Engineering, University of Electronic Science and Technology of China (UESTC), Chengdu, 611731 China; 2 Department of Electrical Engineering and Technology, Government College University Faisalabad, Faisalabad, 38000 Pakistan; 3 Yangtze Delta Region Institute (Huzhou), UESTC, Huzhou, 313001 China; 4 James Watt School of Engineering, University of Glasgow, Glasgow, G12 8QQ UK; 5 School of Electronic Engineering and Computer Science, Queen Mary University of London, London, E1 4NS UK; 6 College of Engineering and IT, Ajman University, Ajman, United Arab Emirates; 7 Electrical Engineering Department, Jouf University, Sakaka, Aljouf 72388 Kingdom of Saudi Arabia; 8 Artificial Intelligence Research Center (AIRC), Ajman University, Ajman, United Arab Emirates

**Keywords:** Electrical and electronic engineering, Engineering

## Abstract

This paper presents a block-chain enabled inkjet-printed ultrahigh frequency radiofrequency identification (UHF RFID) system for the supply chain management, traceability and authentication of hard to tag bottled consumer products containing fluids such as water, oil, juice, and wine. In this context, we propose a novel low-cost, compact inkjet-printed UHF RFID tag antenna design for liquid bottles, with 2.5 m read range improvement over existing designs along with robust performance on different liquid bottle products. The tag antenna is based on a nested slot-based configuration that achieves good impedance matching around high permittivity surfaces. The tag was designed and optimized using the characteristic mode analysis. Moreover, the proposed RFID tag was commercially tested for tagging and billing of liquid bottle products in a conveyer belt and smart refrigerator for automatic billing applications. With the help of block-chain based product tracking and a mobile application, we demonstrate a real-time, secure and smart supply chain process in which items can be monitored using the proposed RFID technology. We believe the standalone system presented in this paper can be deployed to create smart contracts that benefit both the suppliers and consumers through the development of trust. Furthermore, the proposed system will paves the way towards authentic and contact-less delivery of food, drinks and medicine in recent Corona virus pandemic.

## Introduction

INTERNET of Things (IoT) has transformed our lives into world of connect physical objects, and this smart ecosystem has fostered countless innovative applications such as smart retail, connected cars, healthcare, smart automation, smart cities and etc.^1^. Radio frequency identification (RFID) is one of the key technologies enabling the IoT^2^. The ultra-high frequency (UHF) RFID technology is most promising as compared to other competitors (such as low-frequency RFID and high frequency RFID) chiefly because the structures can be easily printed, and provide a relatively longer read range^3,4^ . Such features have made the UHF tags ideal for retail management and other supply chain applications. Despite the wide use, the UHF tags do have their limitations, particularly the sensitivity towards different high relative permittivity tagging surfaces such as metal and other water based substances^5^. The high permittivity and conductivity of liquid fluids make it very challenging to design a tag for such products. This challenge is magnified further due to the low-cost requirements for bulk tagging. To deal with this problem, researchers have worked in the past where a UHF RFID tag antenna was proposed for liquid-filled plastic bottles in^6^. This tag antenna utilized a folded dipole and loop matching technique to get a read range of 4.2 m after mounting it on a plastic water bottle. However, the proposed design was bulky, as the tag had large dimensions and was printed on a non-flexible polyimide substrate. In another work^7^ , a flexible UHF RFID tag antenna was proposed for glass bottle tagging, which was based on loop structure and silicon rubber substrate with dimensions 70 30 3 mm$^3$. This tag achieved a read range of 1.6–2.5 m with glass bottles in self-service grocery stores and other applications. However, the flexible tag antenna was wrapped around the bottle neck, due to which the tag is prone to damage. Another drawback associated with this flexible tag antenna was its large dimension and costly structure. An inkjet-printed low-cost tag solution was proposed in^8^ for tagging liquid plastic bottles by utilizing the equivalent circuit analysis technique. This tag antenna was proposed by modifying the structure of a commercial UHF RFID tag where two parallel L-shaped strips were added to the tag along matching the loop. Although the size of the antenna was not large, it suffered from a relatively small read range, which is not enough for many applications.

A small conformal RFID tag antenna for tagging plastic water bottles was presented in^9^ which had nested-slots integrated with dipole-type RLC strips. This configuration is good enough to provide an impedance match with the RFID chip on the surface of the water bottle. The real part of impedance of tag is very low in free space just 0.1–1 $\Omega$, while on the other hand, it is ranging from 12 to 20 $\Omega$ on the surface of a water bottle which is increased due to increase in loss resistance of the antenna. Moreover, it is observed that the gain of a tag antenna is significantly reduced after mounting it on the surface of the water bottle as compared to the free space. However, the gain of the tag after mounting on the water bottle is enough to give a read range of 3 m. Therefore, the design of tag antenna for water-based surfaces with desired features such as low-cost, long read range, inkjet printable structure, and robustness at mounting position is necessary for various applications like automatic refrigerators, and supply chain management. Recently, characteristic mode analysis (CMA) has been widely employed in antenna design and applications due to its versatility of providing physical insights regarding an antenna’s radiation phenomena^10,11^. As a result, CMA has also been used to design and tune UHF RFID tags for different surface environments^12–14^. In^14^, a vehicle’s license plate was exploited as a RFID tag using CMA. Four coupling slots were created to excite the desired modes. In another study, diagonal and parallel slots were designed using CMA to get a dual polarized structure which resembled a planar pyramid^15^. A circular polarized RFID tag antenna with 45 main beam direction was proposed for metallic poles tagging using CMA^12^. All aforementioned tag antennas were not suitable for water bottles tagging due to their large dimensions and low-cost and bulk tagging requirements of liquid bottles. However, we utilized CMA technique for optimization of proposed liquid bottle tag antenna.

In addition, apart from a tag design (TD) and RFID system, some other technologies such as blockchain are also pivotal for authentication, tracking and timely delivery of products. Blockchain is also a vibrant technology to accomplish IoT dreams^16^. In^17^, a blockchain based IoT architecture was proposed for food supply chain transparency. The complete architecture includes an implementation of the RFID based sensor and blockchain. However, the tag designed in this work is not robust towards different food packaging surfaces. In light of this, RFID and blockchain are considered as dynamic duals for modernizing supply chain and automatic industrial applications.

In^18,19^ , authors have proposed a blockchain based management system to identify fake and counterfeit products, for which the risks can be avoided by integrating proof of possession in the proposed scenario. Moreover, the buyer can verify the authentication and genuineness of product through modified RFID tags and can reject the purchase. We overcome the weakness of the above methodology by implementing the smart contract using the Ethereum blockchain. Our proposed scheme creates trust between the supplier and consumer. Furthermore, in^19^ the authors proposed a trustworthy proof of delivery (POD) mechanism using blockchain by eliminating third party to achieve trust between the buyer and seller. The main problem lies in establishing a trust-based delivery mechanism. Current protocols lack features such as trust, transparency, security and traceability. The proposed scheme fills this gap and creates trust between the parties. For this purpose, we design a smart contract that facilitates by creating trust between the parties, and a mobile application that keeps track of the products in real-time. The process of the supply chain is as follows: i) The RFID tags mounted on each product contains the information about product authentication, delivery location and date. ii) Goods are stored in the blockchain ledger using the RFID reader, it will automatically read the batch information of the goods in the smart supply chain process. iii) The trust between the consumers and suppliers uses blockchain based applications to trace and track the products iv) The buyers can also use blockchain based app for bidding of products and buyer with optimal price will be able to get the contract. The proposed smart contract will then help both parties to track the status and final delivery performance. This work pioneers in proposing a low-cost, reliable, and robust UHF tag antenna based on nested slot configuration^20,21^for liquid bottles with 2.5 m read range improvement over existing designs along with robust performance on different liquid bottle products. Also, we designed a RFID system-based mobile application for enabling smart supply chain process and IoT Applications. Our contribution is listed as follows:

1. We designed a low-cost inkjet-printed UHF RFID tag antenna for item level tagging of hard to tag liquid bottles products that outperforms previous designs.
2. The proposed tag was optimized using characteristics mode analysis.
3. The proposed tag was commercially tested using a conveyor belt and smart cabinet system.
4. We designed an RFID system-based mobile application for enabling the smart supply chain process and Blockchain enabled IoT applications. This app shows a potential to revolutionize the supply chain process by providing the traceability in each stage of the supply chain.
5. Moreover, the smart contract can detect counterfeit items thereby preventing any fraudulent activity.

The rest of the paper is organized as follow: “Secure smart supply chain process for IoT” introduces smart supply process for IoT. “Analysis and design of tag antenna using CMA” provides analysis and design guideline regarding tag antenna design for hard to tag items such as drinks and water bottles. “Parametric study of proposed tag antenna” deals with measurement and simulation results regarding tag performances and read range testing. To validate the proposed concept, “Results and discussion” presents the results of the proposed tag for different liquid bottles. In “Experimental testing” section, results from the commercial testing of the proposed tag for conveyor belt and smart cabinet applications are presented. Finally, Concluding contributions of this research study are provided “Conclusion”.

## Secure smart supply chain process for IoT

**Figure 1:**
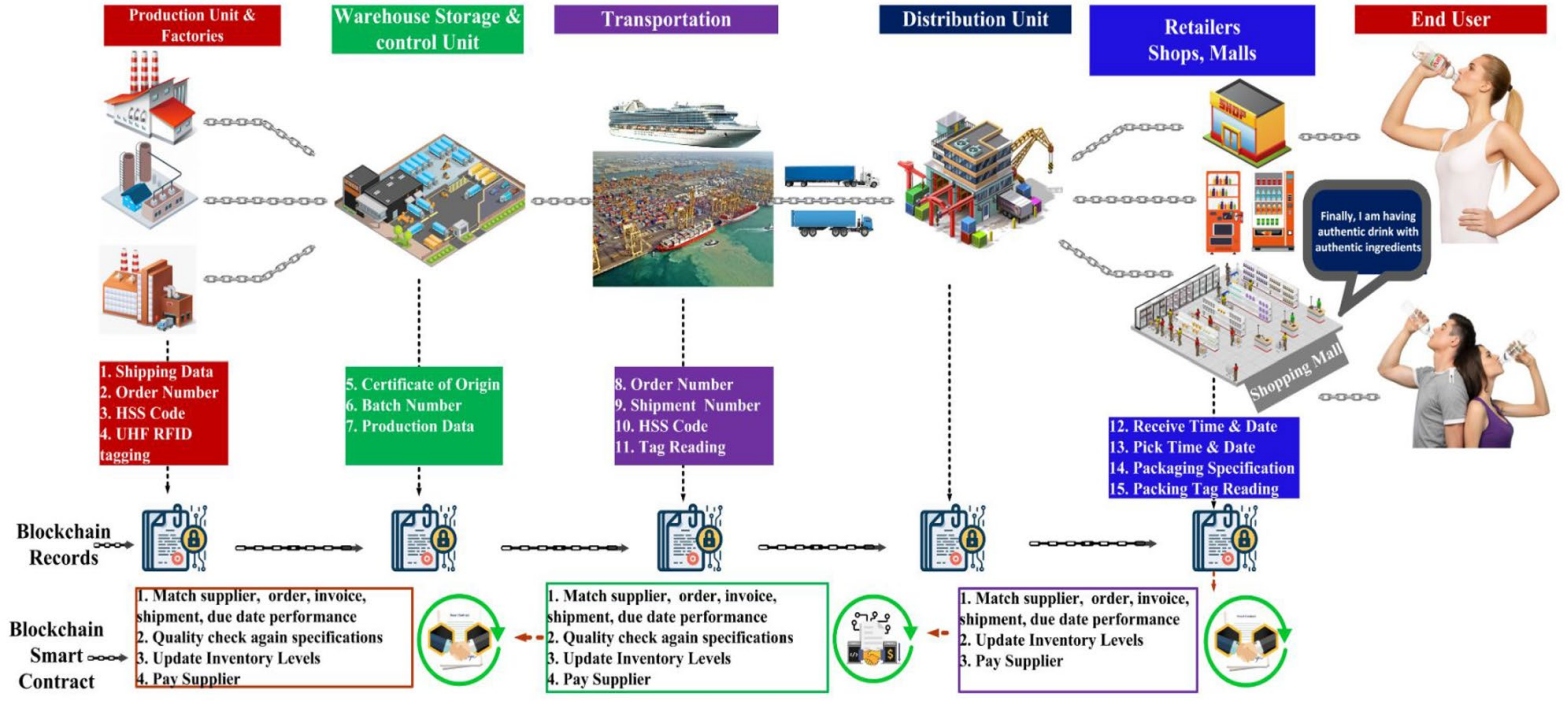
RFID and blockchain based framework for liquid bottle product traceability in the supply chain from manufacturer to retailer. Consumers and suppliers can trace and track the RFID tagged liquid bottle products using blockchain based app.

Our motivation of using blockchain technique is to design a secure communication platform amongst the RFID tag, reader and the supplier in real-world applications. Our proposed scheme provides the security and authentication mechanism based on a blockchain. The blockchain framework provides the product traceability in the supply chain process. Figure 1 shows the architecture of RFID and blockchain based framework for liquid bottle product traceability in the supply chain from manufacturer to retailer. Both the consumers and suppliers can trace and track the RFID tagged liquid bottle products using the developed blockchain based app. The secure system provides information about the product (goods) for the quality and originality of the scheme. Furthermore, RFID tag is attached with each product in supply chain process that enables tracking of the goods in the distribution phase using the decentralized network as shown in Fig. 1.

### Major goals of supply chain process

1. Item Reading: The designed smart contract is able to read the items and register the products in the blockchain ledger.
2. Item Tracking: The blockchain provides the facility to track the items in the whole supply chain (e.g., consumers, buyers, retailers, suppliers, etc.).
3. Verification of Goods: We propose smart contract for updating the transaction of goods. The RFID reader scan all the products and update the new goods information in the blockchain ledger. Thereby, any counterfeiting in product’s expiry date or original contents can be easily detected.
4. The genuineness of the products can be automatically verified using a blockchain ledger.
  The proposed scheme protects and authenticates the goods in supply chain process. Blockchain guarantees the trust, confidentiality, and integrity of the transaction. The blockchain prevents the attacks or modify of data in the supply chain process.

### Components of the blockchain based supply chain

**Figure 2:**
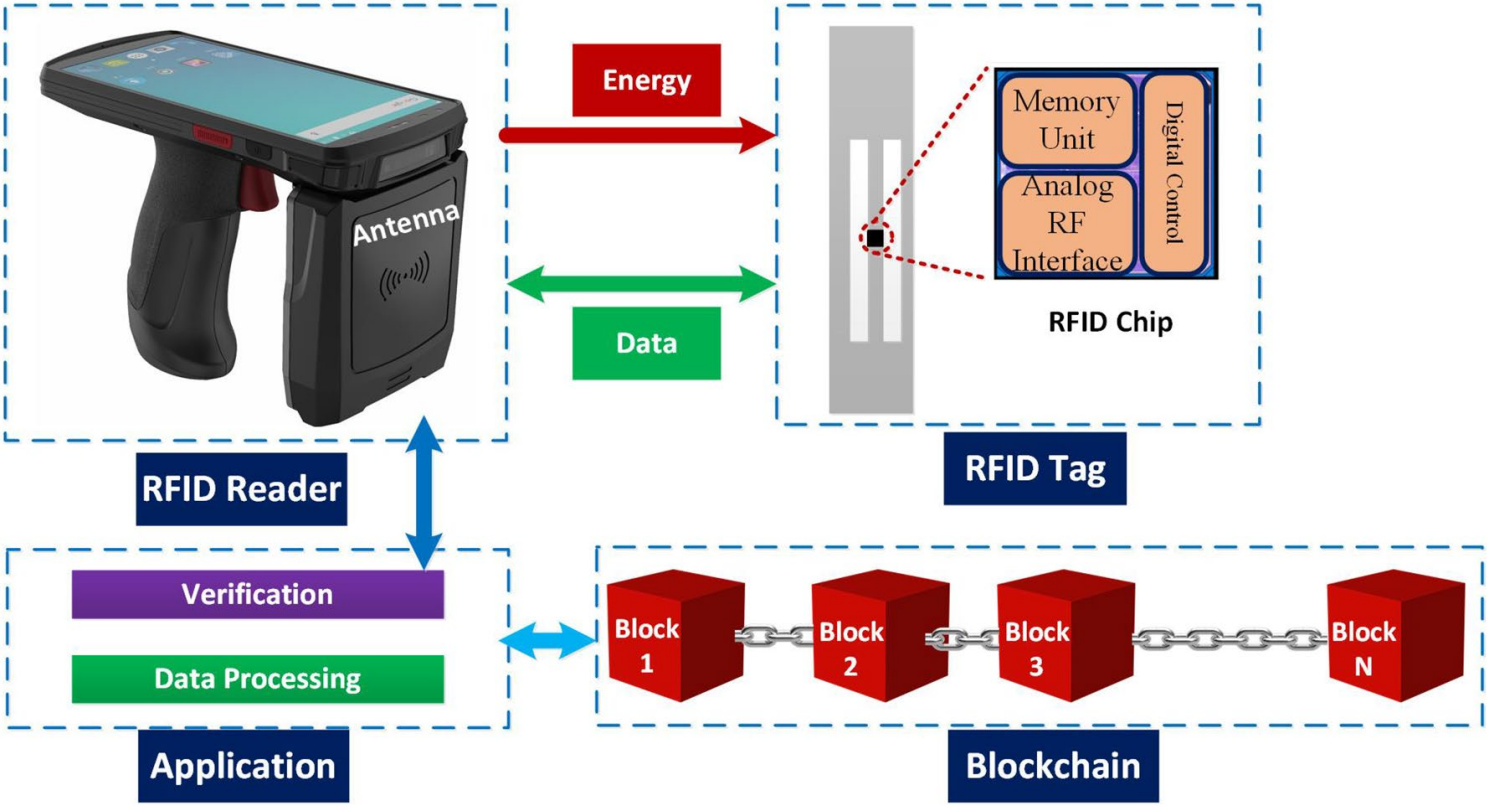
Integration of RFID system with blockchain for supply chain process.

Figure 2 shows the integration of RFID system with blockchain for supply chain process. The RFID reader sends a signal towards tag antenna for data query. The UHF RFID tag uses the reader signal to power the RFID chip, which uses that signal to backscatter the data towards RFID reader. The RFID reader transfers this data to app for data verification and processing. Finally, the app stores and retrieves the data from blockchain through the network.

The components of RFID and blockchain based system for supply chain process are as follows:

1. Blockchain ledger: All goods information (i.e., price, expiry) are stored in the blockchain ledger.
2. RFID Tag: The batch of the product information is stored in RFID tags mounted on each product and the data is stored through the registering the products as a smart contract. For the traceability of the goods, we used the international commodity coding standard EAN/UCC-13. Moreover, RFID tags are scanned at each node of the supply chain of the products such as retailer dealer.
3. RFID Reader: There are two advantages of the RFID reader. (i) UHF reader to add the batch of goods in the blockchain ledger. The range of UHF reader usually ranges up to 10 m. (ii) All goods can track and monitor in process of goods distribution. For example, customer can track the expiry date, contents of food, and delivery of products.
4. Smart Supply chain: In the proposed supply chain we installed the RFID readers to monitor the goods and trace the fraud of goods. As shown in Fig. 1, the process of the smart supply chain and smart contract stores and track the data in each step.
5. Smart Contract: The smart contract provides the authentication mechanism and creates trust among the users and suppliers in the supply chain process. The smart contract provides the security and transparency among the supplier, manufactures, distributors, retailers, and consumers. To trace the products, we design the smart contract for the product registration that registers all the products in the blockchain network. The manufacturers register the product information (i.e., product code, name, expiry-date, etc.) in the distributed ledger. The smart contract updates the transaction in every phase of the supply chain. In the first step, it adds a transaction in the blockchain for the sender recipient and receiver recipient. The product identification code is attached with the previous transaction history. In the second step: customers and Supplier can verify the transaction of the products. Algorithm 1 and 2 implements the smart contract of register the products and updates the transactions in each step, respectively.

In summary, the blockchain prevents the fake RFID tag, misuse of rewriting the orders that pay less, swapping of the RFID tags in the goods, and provides trust between the supplier, manufacture and customers.

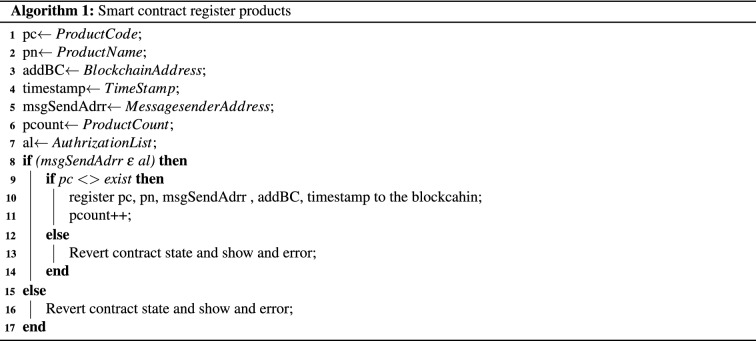

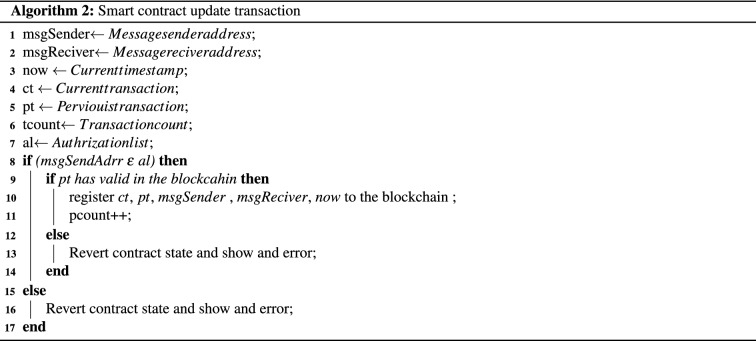





Algorithm 1 demonstrates the implementation of the smart contract regarding the registration of products. Algorithm 2 update the transaction in each step of supply chain process. In Algorithm 1, the RFID reader read the product tags and automatically fetches the product code and product name in the blockchain data base to register the products. The variable Timestamp and blockchainAddress, shows the registerted products time and address of the block. Firstly, the smart contract check the authorization of the manufacturer If(msgSendAddr $\epsilon$ al) from the blockchain ledger. If the authorization is passed, then check the authenticated the product codes if (pc $< >$exist). If the code is find then register the products in the blockchain ledger with the address of the manufacturer msgSendAdrr, product code pc, product name pn, and time stamp. If the authentication is fail, then the fake products or manufacturer is not allowed to save the products in the blockchain ledger. Similarly, In Algorithm 2, update the transaction, when receiver or consumer receive the products then add the new transaction in the blockchain ledger with the previous address of the product and the original manufacturer or supplier of the products. In this way, the smart contract helps to trace the products from the supply chain.

## Analysis and design of tag antenna using CMA

**Figure 3:**
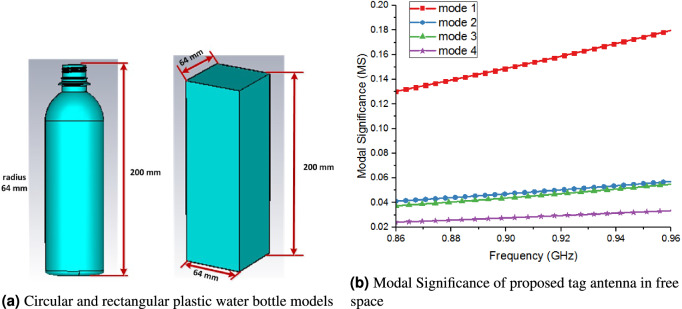
Water bottles models along with modal significance of proposed tag.

As mentioned earlier, water bottles and drinks are most challenging objects from tagging (UHF RFID tags) perspective due to conductivity and high permittivity of water. Therefore, we started with most commonly used UHF RFID tag configurations to find low-cost, robust and optimal solution for water based products and liquid plastic bottles. In this regard, we use two plastic water bottle configurations (bottle material $\varepsilon _r$ = 3.4) as shown in Fig. 3a. The co-design approach was used to simulate the effect of water on different tag configuration. This approach includes the water, plastic water bottle, flexible paper substrate and tag antenna present in one electromagnetic environment. For simulation purposed, the dielectric constant and conductivity of fresh water was taken as 79.5 and 0.01, respectively. Also, the dielectric constant of water bottle and tag’s paper substrate was taken as 3.4 and 3.1 respectively. The CMA technique was used for analysis and design of proposed tag antenna. Characteristics modes (CM) are conventionally defined in terms of eigenvalue equation^22^ as,

1 $$\begin{aligned}{}[X]\overrightarrow{{J_n}} = {\lambda _n}[R]\overrightarrow{{J_n}} \end{aligned}$$

where $\lambda _n$, $\overrightarrow{J_n}$, [X], [R] are eigen values, eigen currents, imaginary, and real parts of the Method of Moments (MoM) impedance matrix, respectively. Modal Significance (MS) is one of the important parameters to describe the eigen values, and further explores the mode resonance behaviour and radiation capacity^22^.

2 $$\begin{aligned} MS = |\frac{1}{{1 + {\lambda _n}}}| \end{aligned}$$

Moreover, MS also paves the way to determine the bandwidth (BW) of each mode even in the absence of external feeding source. According to MS, the half power BW can be defined as:

3 $$\begin{aligned} BW = |\frac{{{f_{upper}} - {f_{lower}}}}{{{f_{res}}}}| \end{aligned}$$

where $f_{upper}$, $f_{lower}$, and $f_{res}$, are the upper band, lower band and resonant frequencies, respectively. The MS value helps to determine the aforementioned frequencies as:

4 $$\begin{aligned} MS({f_{upper}})= & {} MS({f_{lower}}) = \frac{1}{{\sqrt{2} }} \end{aligned}$$

5 $$\begin{aligned} MS({f_{res}})= & {} 1 \end{aligned}$$

**Figure 4:**
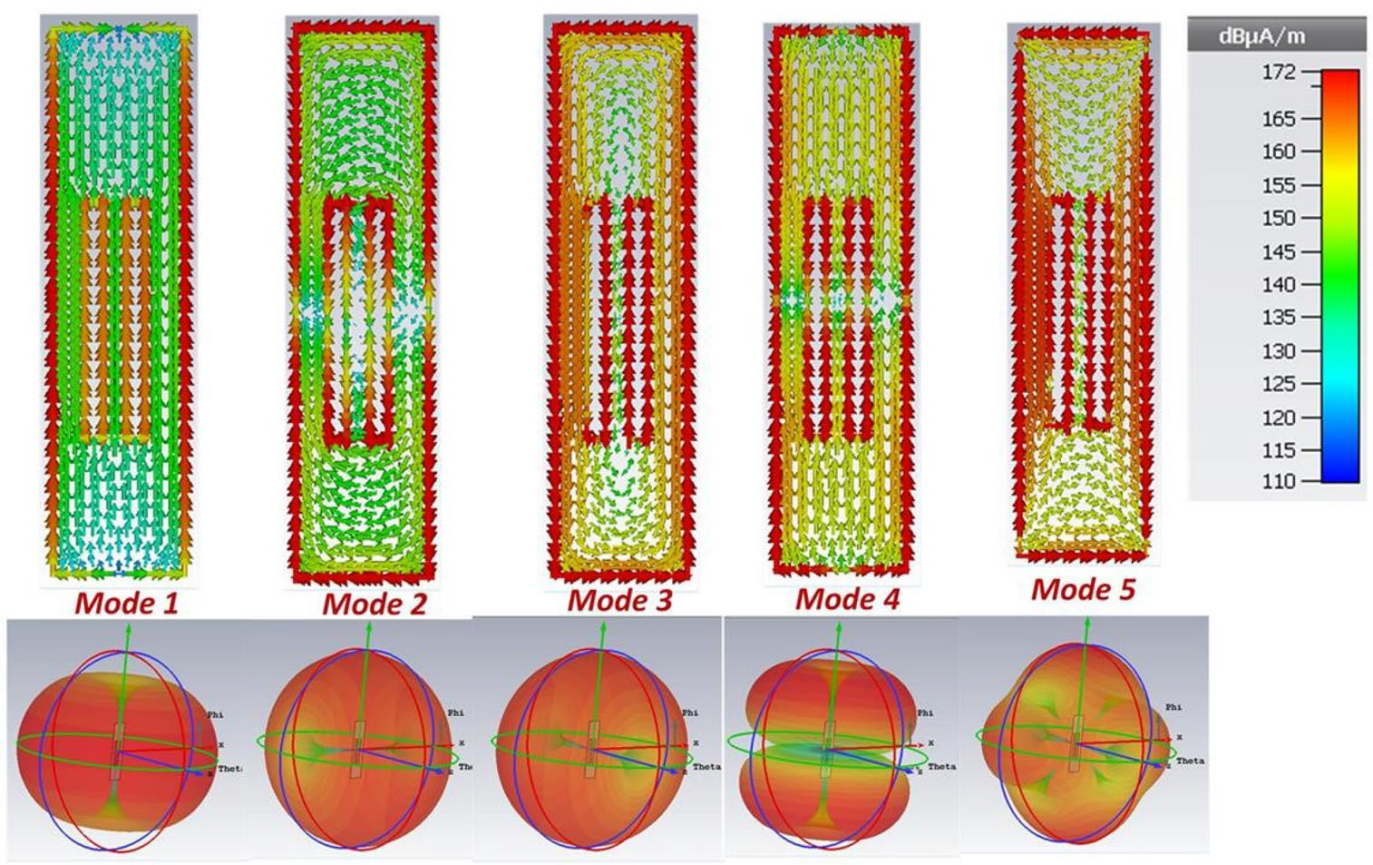
Current distribution and far-field associated with five modes of proposed tag antenna in free space.

CMs with value greater than $1/\sqrt{2}$ are known as significant mode, whereas the ones less than $1/\sqrt{2}$ are non-significant modes. Figure 3b shows the MS of first five modes of the proposed TD in free space. Mode 1 depicts highest value MS 0.16 that expresses the more radiation capacity of mode 1 as compared to other modes in free space. Although mode 1 is non-resonant and non-significant mode in free space, however Fig. 3b gives us clue regarding the radiation capacity of all modes. Additionally, the modes 2, 3, 4 and 5 are also non-significant mode with MS values less than 0.05. Moreover, CM analysis of the proposed tag also gives us an idea about which mode can be used to achieve good performance and read range after mounting of the tag on water bottles. To investigate it further, the CM currents and associated far field of first five modes of the proposed TD are shown in Fig. 4. The mode 1 current depicts the behaviour of dipole like current, more precisely, it represents antenna mode behaviour of folded dipole. For a folded dipole, the two closely spaced currents flow along the conductors’ elements. Therefore, this mode can be regarded as dual double folded dipole mode. Moreover, the horizontal edge produces little currents in mode 1. We can say the main contribution of current was due to vertical edges, rest of the antenna shows a small current value for mode 1.

**Figure 5:**
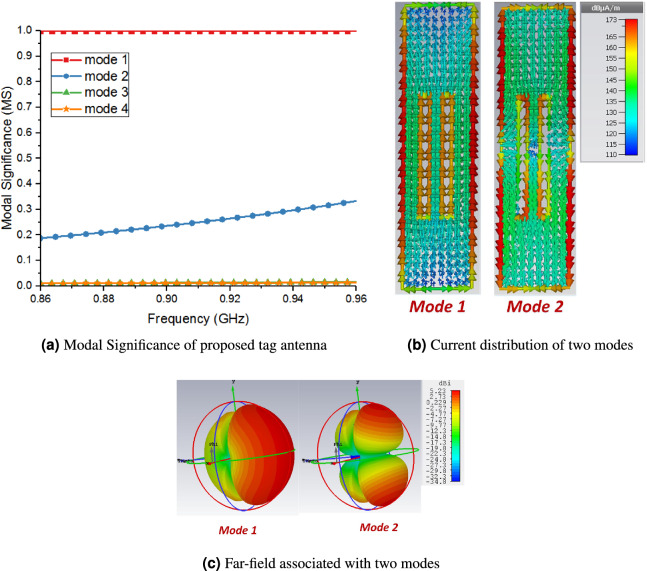
CMA of proposed tag antenna above liquid filled plastic water bottle.

Similarly, mode 1 also depicts dipole like radiation pattern. Modes 2 and 3 mainly depicts loop like surface current distribution, and hence their radiation pattern also resembles that of a loop antenna. Moreover, modes 4 and 5 shows irregular current distribution and their radiation patterns are also complex. Figure 5a shows the MS of proposed tag antenna above a filled plastic water bottle. It can be observed that mode 1 becomes signification mode after placement above the bottle. In order to discuss it further, the surfaces current distribution and associated far-field radiation pattern of proposed tag above water filled plastic bottle are shown in Fig. 5b,c, respectively. The surface current distribution of mode 1 is almost similar to the case of free space. Indeed, the mode 1 continues its non-resonant behaviour above high permittivity materials such as liquid filled plastic water bottle.

**Figure 6:**
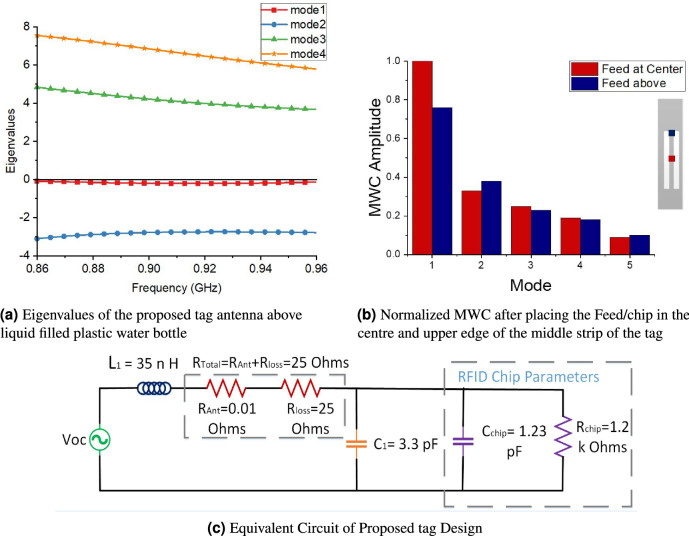
Performance analysis of proposed tag antenna above liquid filled plastic water bottle.

Figure 6a shows the eigenvalue plot of the proposed tag above water bottle which further demonstrates the non-resonant behaviour of mode 1, which forces it to offer inductive reactance to counter the capacitive impedance. Precisely, mode 1 shows non-resonant behaviour in case of both free space and above water bottles. However, this non-resonant behaviour helps to maintain the inductive reactance even above high permittivity surfaces such as water bottles. The current distribution of mode 2 depicts two small antipodal dipoles type radiation pattern and therefore, shows different far-field segments. As CM form an orthogonal set of functions, so, the total current on the surface of the antenna or radiating object can be expressed as the linear superposition of these characteristic mode currents as:

The product provides information about the coupling between the nth order mode and excitation. Furthermore, it also gives information, which mode will be excited by the external feed or excitation source. The choice of the optimal feed position for the tag was explored further using MWC. Figure 6b shows the normalized MWC amplitude after placing the feed/chip position at the centre and upper edge of middle strip of the proposed tag. It can be seen that the normalized MWC has maximum value for mode 1 with feed/chip at centre, which shows that mode 1 excites more efficiently with this configuration. Moreover, if we place the chip/feed at the upper edge of middle strip of tag, although the normalized MWC is higher for mode 1, however this value is less as compared to the MWC value for centre feed position. Consequently, the centre feed/chip position is the optimal feed location to excite mode 1 efficiently with more mode purity. The equivalent circuit parameters of the proposed tag were estimated by applying electric wall concept^23^ and simulated using ADS software 2011 as shown in Fig. 6c. These parameters values were optimized by fine-tuning in ADS and further processed in MATLAB 2015a, respectively. These values provide us an understanding of the tag operation to counter the water effects during its operation after mounting above a water filled plastic bottle. This TD provides imaginary part of impedance both in free space and above water bottles. However, the real part of impedance of tag antenna increases over water surfaces due to increase in loss resistance of antenna that gives us good match with RFID chip especially after mounting the tag on water bottles.

## Parametric study of proposed tag antenna

**Figure 7:**
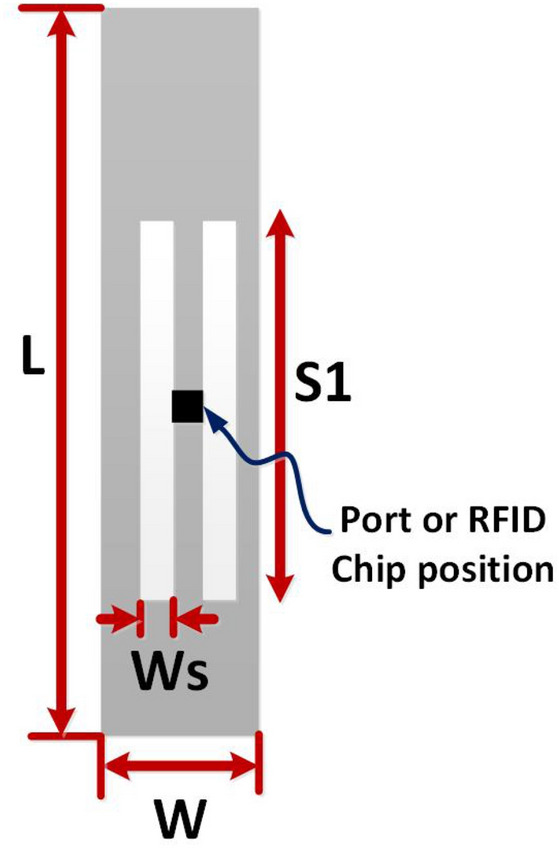
Different parameters of proposed tag design.

**Figure 8:**
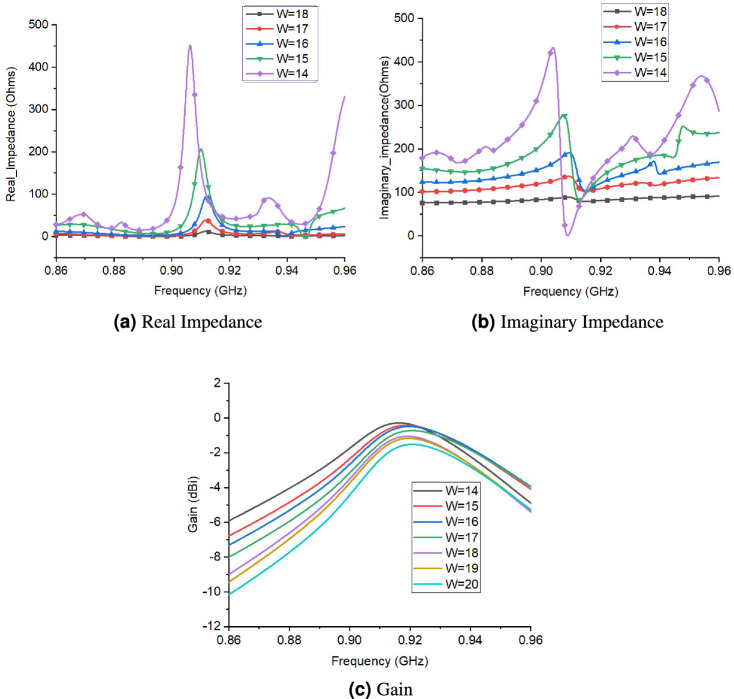
Performance of proposed tag design for different values of W (width of tag).

**Figure 9:**
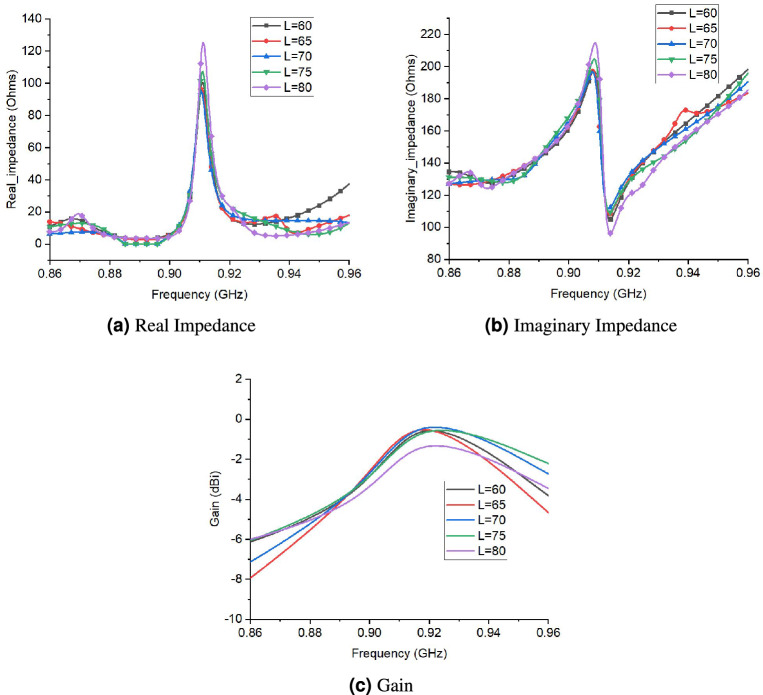
Performance of proposed tag design for different values of L (length of tag).

The parametric study of proposed tag antenna was conducted for rigorous analysis. Figure 7 shows different parameters of proposed tag design. Figure 8 presents the performance of proposed tag antenna for different values of W (width of tag). The real impedance of tag antenna decreases as width of tag increases. Similarly, Fig. 8b shows variations of imaginary impedance against different values of W. This potentially indicates that increasing the W will decrease the impedance match and bandwidth. Figure 8c shows the gain of tag antenna with different values of W. The gain of tag antenna decreases with increase in W as it increases the are under influence of liquid. So, W=15 is chosen as optimum value of width. The performance of tag antenna for different values of L (length of tag) is shown in Fig. 9. Figure 9a,b shows the effect of the changes in L on real and imaginary impedance, respectively.There is slight increase in value of real and imaginary impedance values as L increases from 60 to 80 mm. The effect of L on gain is depicted in Fig. 9c, that indicates very small variation in gain except for L=80,where there is decrease in gain value is observed.

**Figure 10:**
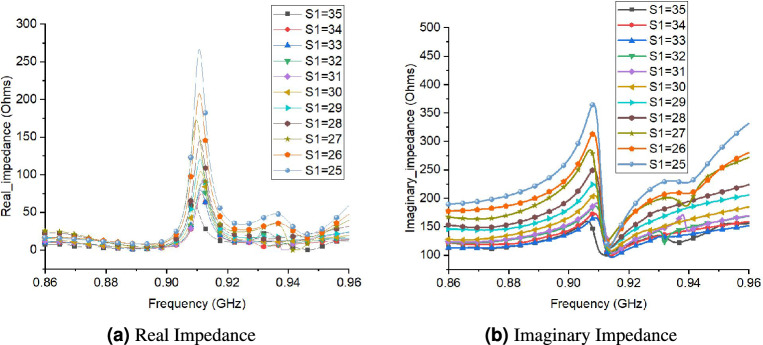
Performance of proposed tag design for different values of S1 (length of slots).

**Figure 11:**
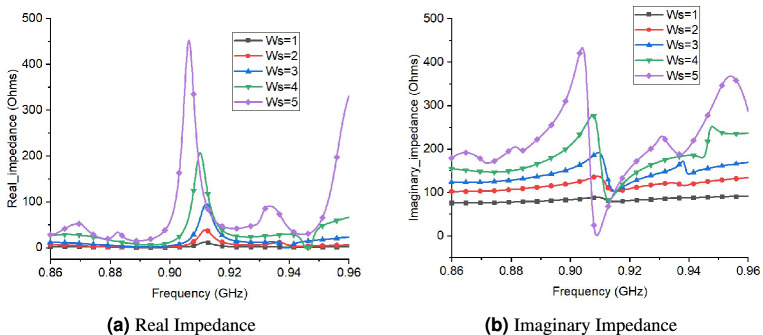
Performance of proposed tag design for different values of Ws (width of slots).

Figure 10 shows the effect of S1 (length of slot) changes on real and imaginary impedance. There is a decrease in both the value of real and imaginary impedance for increasing value of S1. Consequently, by increasing the slot length, the bandwidth of the antenna decreases. The optimal value chosen for S1 is 29 to get impedance match with RFID chip. Moreover, the effect of Ws (slot width) on performance of tag is shown in Fig. 11. The real and imaginary impedance increases significantly with increase in value of Ws (slot width). This indicates that the bandwidth of tag antenna decreases with increase in value of Ws. The optimum value chosen for Ws is 3.

## Results and discussion

**Figure 12:**
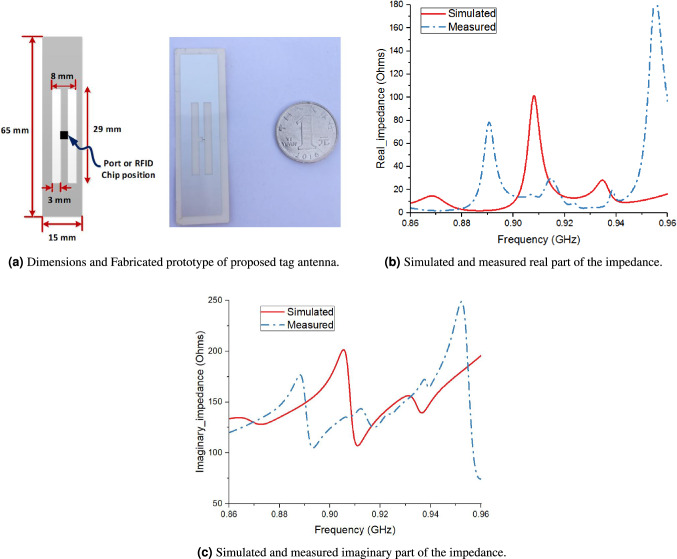
Performance of proposed tag antenna after mounting on a circular water filled plastic bottle using full wave simulation in CST Microwave 2018.

Figure 12a shows the dimensions of the tag antenna that has been optimized for impedance matching with a commercially available RFID chip, Impinj Monza R6. As can be seen in Fig. 12a , the tag antenna was fabricated on a 100 m paper substrate using an ink-jet printer having conductive ink (15 m silver with $\sigma$ = 12.5 ${10^6}$ S/m). The real and imaginary parts of the simulated and measured impedance of proposed tag antenna after mounting on a water filled plastic bottle are shown in Fig. 12b,c, respectively. The simulated impedance was obtained after placing the tag antenna both at a rectangular water bottle and circular water bottle with dimensions 200 64 64 ${mm^3}$ as shown in Fig. 3a. The material of simulated water bottle was polyethylene terephthalate (PET) with relative permittivity, $\varepsilon _r$ = 3.4, loss tangent, $\tan \delta$ = 0.002, and thickness, h equal to 0.3 mm. Moreover, to obtain more robust results, the $\varepsilon _r$ and h were varied from 2.9 to 3.4 and 0.2 to 0.5, respectively. The simulated parameters of water were taken as $\varepsilon _r$ = 78 and $\sigma$ = 0.01 S/m)^24^, however these parameters for tap water were listed as $\varepsilon _r$ = 79.2 and $\sigma$ = 0.267 S/m (at 1 GHz) at $20\,^\circ$C. The simulated real part of impedance of the proposed tag antenna over water filled plastic water bottle surface ranged from 25 to 14.5 $\Omega$ in part of US RFID frequency band (910–928 MHz), with an exceptional behaviour from 900 to 910 MHz where the real part of the impedance reaches a peak value of 100 $\Omega$. The tag impedance was measured by employing the inlay without RFID chip. The impedance is measured using a vector network analyser through a balance to unbalance converter (balun). The real part of the measured impedance ranged from 8 to 25 $\Omega$. The difference between the simulated and measured impedance is attributed to the difference in material properties of water. Also, it can be observed that the simulated imaginary part of impedance of the proposed tag over water bottle surface ranges from 150 to 200 $\Omega$, whereas the measured imaginary part lied in the range 125–150 $\Omega$. The simulated as well as the measured impedance values give a good impedance match with Impinj Monza R6 RFID chip. The impedance of R6 RFID was estimated using ADS by considering its parameters Cic = 1.23 pF and Ric = 1.2 k$\Omega$ provided in the datasheet^25^ with impedance value of 16–140 j $\Omega$ at 915 MHz. Although the impedance measurements method that uses a balun is known to have accuracy issues, we obtained satisfactory performance out of the proposed tag antenna in US RFID band, which was further verified from a more accurate read range measurement method using the Voyantic Tagformance pro setup, which is discussed in detail later in this section.

## Experimental testing

**Figure 13:**
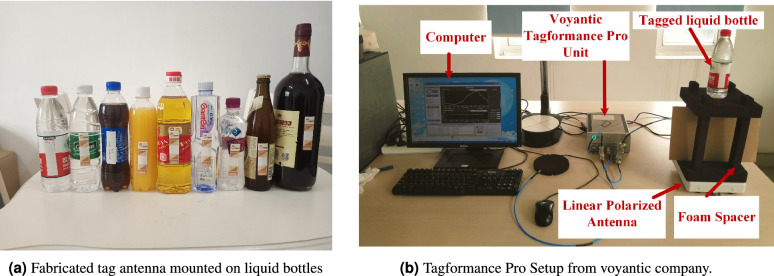
Measurement set for read range of proposed tag after mounting on different liquid bottles.

Although the tag antenna was designed and optimized with an aim to work on water filled plastic bottles, it yielded satisfactory performance on other bottles with different sizes, materials, and liquids. To prove the use of the proposed tag on different bottles, the fabricated tag antennas were pasted on three different plastic (cola, mango juice, and non-steam soda drink, cooking oil), and glass bottles (beer, and red wine) as shown in Fig. 13a. The performance of proposed tag antenna was measured in terms of read range after mounting the tag antenna on these bottles (as depicted in Fig. 13b) using Voyantic Tagformance Pro which consisted of a linearly polarized antenna with 6 dBi gain, a foam spacer with 30 cm length, and a computer with software setup installed for read range measurement. The setup worked by sending input signals with known transmitted power to the tag antenna. The tag antenna received a portion of the transmitted power (also known as received power) due to power loss in cable and free space. The RFID chip utilized the received power to send backscattered signals back towards the measurement setup. Accordingly, the resonant frequency and theoretical reading range of the tag antenna were determined. Figure 13b shows the read range measurement procedure of the proposed tag antenna after mounting on different bottled products using the Tagformance setup. The read range of tag antenna was measured both after placing the bottles on foam spacer in the downward as well as in the face to face directions.

**Figure 14:**
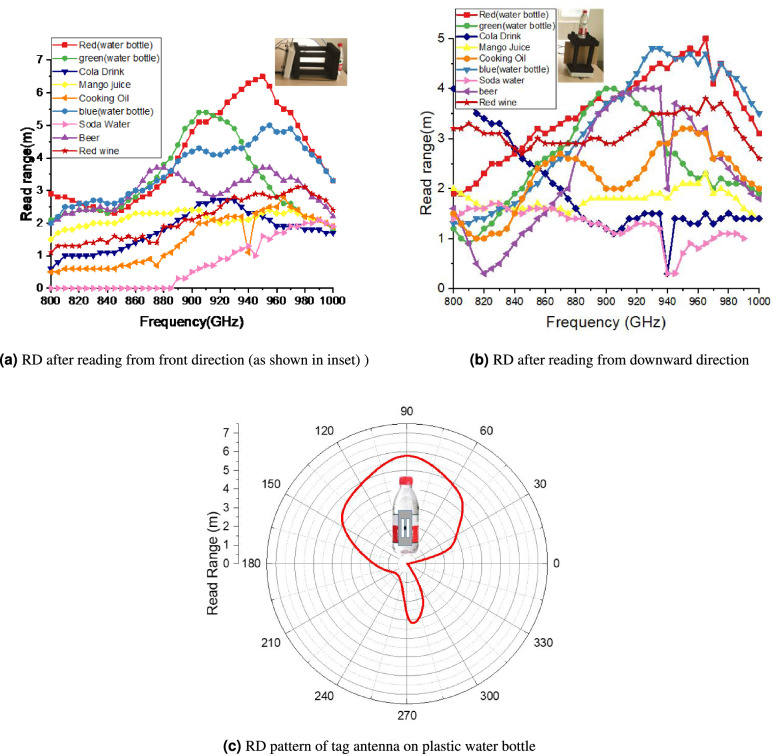
Read range (RD) of proposed tag antenna after mounting on different liquid bottle products.

**Figure 15:**
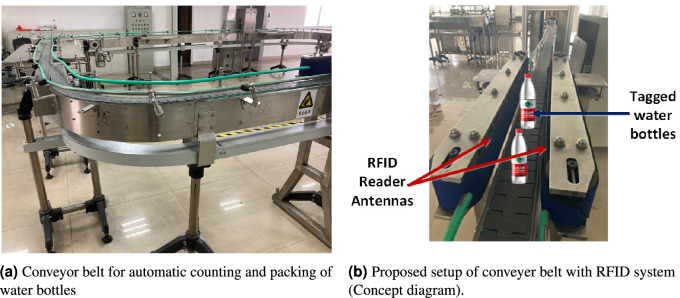
Conveyor belt based commercial application using proposed tag based system.

**Figure 16:**
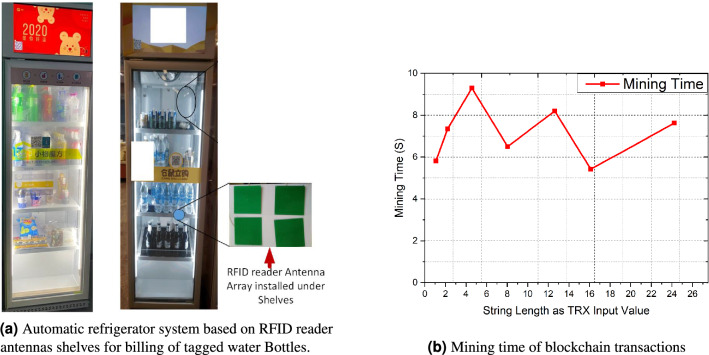
Integration of automatic refrigerator system for billing of tagged water bottles.

Figure 14a shows the read range of the proposed tag antenna after mounting on different liquid bottle products from front direction (configuration in the inset). The proposed tag shows a maximum read range of 6.5 m from front direction on a water bottle whereas the least read range of approximately 1 m on a soda drink that may be due to the high sodium bicarbonate content of 35mg/100 mL, which increases the conductivity of water. The proposed tag antenna depicts the read range (from front direction) of 2.6 m, 2 m, 2.1 m, 2.7 m and 2.6 m after mounting on cola, mango juice, cooking oil, beer and red wine bottles, respectively. Although the tag was optimised for the US RFID band, it works equally well in the European RFID band (ETSI Lower Band 865–868 MHz) and EU upper band (915–921 MHz) as well. To prove the robustness of the proposed tag antenna, the read range was also recorded in the downward direction as shown in Fig. 14b. The read range in the downward configuration is more than 4 m on three water bottles with a maximum read range of 5.2 m on a spring water bottle. The proposed tag antenna depicts the read range (from downward direction) of 1.5 m, 1.8 m, 2.7 m, 4 m, 3.2 m and 1 m after mounting on cola, mango juice, cooking oil, beer, red wine bottles and soda drink, respectively. Figure 14c shows the read range pattern of tag antenna on plastic water bottle. The read range pattern is looks like directional antenna,however,it shows one exception reading from back side similar to dipole like tags. The proposed tag antenna was used for conveyor belt based commercial application for automatic counting, and packing of water bottles as shown in Fig. 15. The antipodal dipole based long RFID reader antennas were installed on sides of conveyor belt for reading of water bottles running on belts. Moreover, the proposed tag antenna was also tested by placing the tagged water bottles in commercially deployed automatic refrigerator based system. The automatic refrigerator system was based on RFID reader antenna shelves that contribute toward reading the tagged water bottles as shown in Fig. 16a. The automatic billing experiment was done by placing 30 water bottles and 10 beer glass bottles.

The automatic billing experiment was done by placing 30 water bottles and 10 beer glass bottles. After repeating the experiment, 95 % of the bottles are correctly identified and billed correctly. This experiment shows an accuracy of 95 % yield for automatic billing that proves the robustness of proposed tag antenna. The accuracy can be further improved by carefully fabricating and pasting of the tag antenna. The automatic billing experiment was repeated three times and we found the accuracy to vary from 92.5 % to 97.5 %. Therefore, this proposed tag antenna is a viable potential candidate for supply chain of liquid bottle products and industrial IoT 4.0 applications.

Figure 16b shows how the difficulty level changes with the time for mining of each block when the block is created in the blockchain distributed ledger the mining time for each block is different for each other. The relationship between the input transaction length and processing time depends on the duration of the string length. However, a longer string size arises results naturally in a longer the transaction time. It does not rely on the number of lines of code. Sometimes the mining time performance is not the same due to large input length. Precisely, the amount of gas increase when the input length of the string is increased. Moreover, the truffle server executes the smart contract and cost for each transaction. The cost will be calculated as shown in Table 1, which refers to the gas used for product registration and updating of smart contract. Smart contracts measure cost through gasoline prices. After executing the smart contract, gas 1180217 will be recorded for product registration. This means that the transaction cost of executing a smart contract is $\$$ 0.6987.

**Table 1:** Smart contract cost.

Contract create	Gas used	USD	Actual cost (either)
Product registration smart contract	118 0217	0.6987	0.002360435
Update transaction smart contract	638125	0.3778	0.001276250

**Figure 17:**
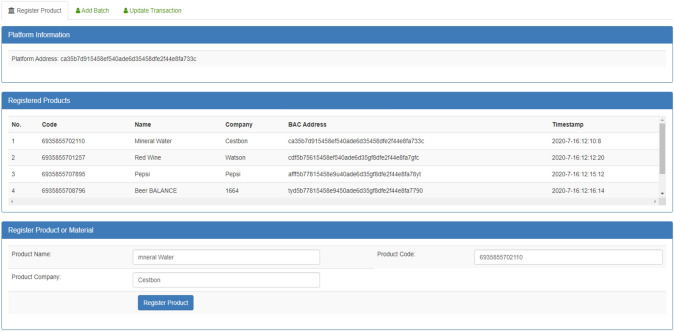
The registration process of the products on blockchain based App.

Figure 17 shows the registration process of the products such as soft drinks (Pepsi, coca cola), beer, on blockchain based App. The companies register their products in to the decentralized blockchain database for create the immutable system. Moreover, the companies can add the product code, product name, etc., by clicking the Register Product button. Also, the smart contract was executed when press the Register Product button. The code of the product follows the standard EAN/UCC-13 scheme for generation the 13-digit code. In addition, this page also provides the facility to add authorized users.

**Figure 18:**
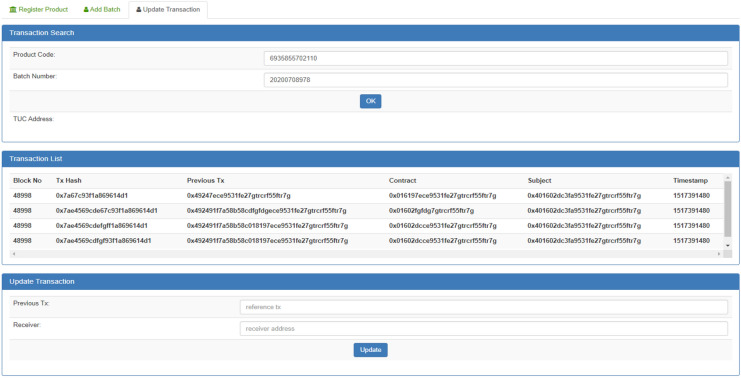
The blockchain transaction record and histories of the updated products.

Figure 18 shows the blockchain transaction record and histories of the updated products which is transfer to the consumers’. The smart contract provides the facility to transfer the funds by checking the authorization of the users. End users can check all transaction by inputting the code and clicking the OK button. Finally, this blockchain based App provides the security/ authenticity regarding smart supply chain process and further create trust between the consumers or providers.

Table 2 shows a comparison of the proposed RFID tag antenna with state of the art. The tag antenna design in^6^ covers 5.6 m read range, however, it is etched on costly polyimide substrate. Moreover, the read range measured was only for one plastic bottle. It’s performance for other water bottles is not verified for robust set of water bottle of different sizes, shaped and material.The tag antenna presented in^7^ exhibited a read range of 1.6–2.5 m on glass bottles with 3 mm substrate.Similarly,the tag designed in^8^ achieved a read range of 0.54 m and 0.31 m on plastic and glass bottles, respectively. The tag proposed in^9^ demonstrated a read range of 7 m on small plastic water bottle,however,it has large dimensions with 0.8 mm substrate, which does not support low cost tagging up to item-levels.Therefore,the novelty of the proposed tag design is compared in terms of cost, size and maximum read range on water bottles as well as other liquid bottles. The proposed tag design achieved a read range of 6.5 m and 2.6 m on plastic and glass bottles containing water.Similarly, the proposed tag design exhibited a promising results regarding read range of approximately greater than 2 m on plastic bottles containing different liquid and drinks such as soft drinks,beer,juice and etc. So, this tag design shows superior performance in terms of read range improvement along with advantage of low-cost and direct printing capability on water bottle surfaces using inkjet printing technology,which is pivotal for item-level or bulk tagging.

**Table 2:** Comparison of proposed tag antenna with state of the art.

Condition	This work	^6^	^7^	^8^	^9^	^10^
Tag size (mm$^2$)	65 15	44 20	70 30	72 30	87.8 57.9	120 30
Thickness (mm)	0.1	0.2	3	0.05	0.8	1.5
Substrate type	Paper	Polyimide	Silicon rubber	PET	Rigid substrate	FR-4
Fabrication cost	Low, inkjet printed	High	High	Low	High	High
Operational bands	EU, US	US	NA	EU, US	US	Very small
Directly printable	Yes	No	No	No	No	No
RD Frontside	6.5 (plastic)	5.6	NAP	0.54	7	2.5
RD Backside (m)	5 (plastic)	NA	NA	NA	NA	NA
RD on glass bottles (m)	2.6	NA	1.6–2.5	0.31	NA	NA
RD on other liquids (m)	>2	No	No	No	No	No
Actual ink area (${mm^2}$)	795	850	NAP	1154	NAP	329.4
RFID chip sensitivity (*dBm*)	– 20	– 20	– 19.5	– 20.5	– 19.5	– 15

$^a$ Read range (RD), $^b$ NA stands for Not Available, $^c$ NAP stands for Not Applicable.

## Conclusion

In this paper, a standalone tag antenna system which is equipped with blockchain technology for a secure, robust and real-time supply chain management, traceability and authentication of hard to tag items such as liquid bottle products. In this context, we proposed a novel, low-cost inkjet-printed UHF RFID tag antenna design with superior performance on liquid bottle products. The nested slot-based antenna configuration achieved a good impedance match on high permittivity surfaces such as liquid bottle products. Moreover, the proposed tag was commercially tested for tagging liquid bottle products in conveyer belt and smart refrigerator systems for automatic billing applications. The RFID reader antenna was placed under the shelf of the smart refrigerator system which automatically read and processed the information stored in the tag regarding products. We also designed an RFID system based application for enabling smart supply chain process. The RFID tags and readers were used to register and track the products in the smart supply chain process. Therefore, the proposed UHF RFID tag assigns a unique identity to items which is essential whilst monitoring products such as liquid bottles in real-time. Finally, we deployed the developed app in a real-world scenario using smart contract. Using the proposed technology, a smart contract can be developed which we hope will foster trust between the consumers and the suppliers. Additionally, this kind of system is very useful in recent Corona virus pandemic situations for secure and contact-less delivery of food, drinks and medicines.
